# Simulation‐based assessment in laparoscopic urology: Model design and validation challenges

**DOI:** 10.1111/bju.70288

**Published:** 2026-04-13

**Authors:** Arianna Pischetola, Willem M. Brinkman, Juan Gómez Rivas

**Affiliations:** ^1^ Faculty of Medicine University Campus Bio‐Medico Rome Italy; ^2^ Department of Oncological Urology University Medical Centrum Utrecht Utrecht The Netherlands; ^3^ Department of Urology Hospital Clínico San Carlos Madrid Spain

**Keywords:** Simulation training, laparoscopy/education, urology training, urological surgical procedures, validation, models, educational

**Abbreviation**
ESU: European School of Urology

Simulation‐based training has become a central component of urological education, driven by patient safety imperatives, reduced operative exposure, surgeon burnout, and the transition from time‐based to competency‐based training models [1]. In Europe, this shift has been operationalised through structured initiatives such as the European Basic Laparoscopic Urological Skills (E‐BLUS) programme and subsequent standardisation efforts led by the European School of Urology (ESU), which emphasise objective assessment and portability across training locations [2, 3]. As simulation increasingly informs progression and credentialing decisions, attention has moved beyond simulator availability towards the validity and interpretability of simulator‐derived assessment scores. Contemporary validity frameworks emphasise that assessment scores must be supported by coherent evidence for their intended use, including content relevance, internal structure and reliability, and relationships to external measures. Within surgical simulation, this re‐frames validation away from demonstrating realism alone and towards determining whether observed performance metrics can meaningfully discriminate levels of expertise and inform educational decisions. However, in practice assessment approaches remain heterogeneous, particularly beyond basic skills training, with substantial variability in model design, outcome metrics, and reporting standards as task complexity increases across training levels.

Effective simulation‐based assessment requires models capable of accurate and reliable measurement. Across contemporary laparoscopic simulators, functional fidelity, that is, features that directly task execution, appears to be more influential than surface realism. Design elements such as realistic tissue–instrument interaction, visual cues for bleeding or leakage, and stable camera perspectives are consistently associated with improved discrimination of technical performance. Model geometry and materials are most effective when informed by task analysis of critical procedural steps and common errors, allowing graded progression, reuse, and feasibility within constrained training environments [4, 5].

Measurement strategies underpin assessment credibility. Most simulation‐based assessments combine process metrics, such as completion time or motion efficiency, with variable inclusion of product‐based outcomes that better reflect clinical priorities, including haemostasis, repair integrity, and precision of closure. While automated capture through virtual platforms or video‐based tracking can enhance reproducibility, automation alone does not compensate for poorly defined metrics or inconsistent scoring frameworks. Standardised tools, including task‐specific checklists and anchored global rating scales, remain essential for converting observation into reliable scores. Importantly, rater reliability should be treated as a design requirement rather than a *post hoc* statistic, particularly when assessment outcomes inform progression.

To ground these methodological considerations in empirical observation, we examined prospectively collected performance data from two intermediate laparoscopic simulation modules, partial nephrectomy and major vessel injury, delivered during ESU hands‐on training courses as part of the Laparoscopic Urological Skills Level 2 (LUSs2) curriculum. These modules were selected to explore how commonly used assessment metrics behave at an intermediate procedural level and whether they discriminate between expert and trainee performance in practice. A total of 27 participants completed partial nephrectomy (4 experts, 23 residents) and 41 completed major vessel injury (29 experts, 12 residents). Experts were fully trained urologists with substantial laparoscopic experience, whereas residents were urologists in formal training. Pre‐defined metrics included procedure completion time and domain‐based qualitative indicators (tissue handling, haemostasis, closure integrity, procedural completeness). Completion times were compared using the Mann–Whitney *U* test, whereas qualitative variables were recorded categorically and summarised descriptively. Across both modules, experts completed tasks notably faster than residents (median [interquartile range] time for partial nephrectomy of 21:36 [17:49–26:21] min for experts vs 42:00 [36:35–43:26] min residents; and for major vessel injury: 5:24 [4:57–8:04] min for experts vs 16:48 [11:12–27:15] min for residents) and demonstrated consistently superior tissue handling, haemostasis, and closure quality. These findings align with prior reports from basic laparoscopic skills programmes, confirming that time‐ and error‐based metrics retain discriminatory value as task complexity increases.

Importantly, exploratory *post hoc* analysis suggested that completion time alone was insufficient to characterise performance quality at this level. Domain‐based scoring incorporating qualitative measures, such as tissue handling, haemostatic control, and procedural completeness, more closely reflected expert performance patterns than speed alone. These observations informed the development of candidate domain‐based scoring frameworks with provisional competency thresholds, intended to guide future validation studies rather than define definitive certification standards. As such, the data extend existing findings by illustrating how assessment metrics behave when applied to whole‐task, intermediate laparoscopic simulations.

Preliminary observational feedback from the advanced step of the ESU Laparoscopic Urological Skills curriculum (LUSs3) provides convergent support for a stepwise approach to simulation design and assessment. In multicentre cadaveric training settings, participants consistently reported high perceived realism and educational relevance of the model for advanced laparoscopic tasks, with stable evaluations across course editions. These observations suggest that, at higher levels of procedural complexity, anatomical fidelity and contextual realism may become increasingly important complements to performance‐based metrics, extending the principles observed at intermediate level without implying definitive validation.

The transferability of simulator‐derived performance to the operating room remains a critical consideration. Evidence from basic laparoscopic tasks suggests that structured error counts and motion economy correlate with intraoperative efficiency and fewer technical errors. However, associations weaken for complex procedures, where anatomical variability, cognitive load, and team dynamics limit direct transfer. These observations reinforce the need for prospective studies linking simulator performance to intraoperative and patient‐centred outcomes, using standardised task definitions and blinded, centralised scoring to reduce inter‐site variability.

Many limitations in the simulation literature are methodological rather than conceptual. Over‐reliance on time‐to‐completion risks reward speed at the expense of safety and technical quality [6]. Heterogeneous error definitions, locally derived scoring rubrics, and single‐rater designs undermine comparability and obscure reliability, particularly when assessment outcomes inform progression decisions [7]. Inconsistent reporting of inter‐rater agreement, internal structure, and test–retest stability further limit interpretability and portability across centres.

Collectively, these observations highlight the need for standardised, purpose‐designed assessment frameworks that prioritise functional fidelity, multi‐domain performance metrics, and transparent reporting of reliability [8]. Proficiency‐based progression, in which advancement is contingent on demonstrated competence rather than exposure time, appears better aligned with both educational efficiency and patient safety. Emerging advances in automation, phase recognition, and skill classification offer the potential to support more objective and scalable assessment, particularly when combined with shared task frameworks and open benchmark datasets.

In summary, simulation‐based assessment offers a scalable pathway towards objective evaluation of laparoscopic competence, but its educational impact depends on methodological consistency and psychometric rigour. Preliminary observational data from intermediate and advanced laparoscopic modules suggest that domain‐based assessment may better capture performance quality than time alone, supporting ongoing efforts to refine validation strategies. These findings add incremental evidence to the evolving simulation literature and may inform the design of future validation studies within competency‐based urological training.

## Author Contributions

Arianna Pischetola: literature search, writing – original draft; Willem M. Brinkman: literature search, writing – review and editing; Juan Gómez Rivas^:^ supervision, writing – editing.

## Disclosure of Interests

The authors declare no conflicts of interest.
